# Potential Role of Synaptic Activity to Inhibit LTD Induction in Rat Visual Cortex

**DOI:** 10.1155/2016/1401935

**Published:** 2016-12-06

**Authors:** Matthew R. Stewart, Hans C. Dringenberg

**Affiliations:** ^1^Department of Psychology, Queen's University, Kingston, ON, Canada K7L 3N6; ^2^Center for Neuroscience Studies, Queen's University, Kingston, ON, Canada K7L 3N6

## Abstract

Long-term depression (LTD), a widely studied form of activity-dependent synaptic plasticity, is typically induced by prolonged low-frequency stimulation (LFS). Interestingly, LFS is highly effective in eliciting LTD* in vitro*, but much less so under* in vivo* conditions; the reasons for the resistance of the intact brain to express LTD are not well understood. We examined if levels of background electrocorticographic (ECoG) activity influence LTD induction in the thalamocortical visual system of rats under very deep urethane anesthesia, inducing a brain state of reduced spontaneous cortical activity. Under these conditions, LFS applied to the lateral geniculate nucleus resulted in LTD of field postsynaptic potentials (fPSPs) recorded in the primary visual cortex (V1). Pairing LFS with stimulation of the brainstem (pedunculopontine) reticular formation resulted in the appearance of faster, more complex activity in V1 and prevented LTD induction, an effect that did not require muscarinic or nicotinic receptors. Reticular stimulation alone (without LFS) had no effect on cortical fPSPs. These results show that excitation of the brainstem activating system blocks the induction of LTD in V1. Thus, higher levels of neural activity may inhibit depression at cortical synapses, a hypothesis that could explain discrepancies regarding LTD induction in previous* in vivo* and* in vitro* work.

## 1. Introduction

It is now widely recognized that the acquisition and storage of information require long-lasting modifications (i.e., plasticity) of synaptic transmission among neurons [1–3]. Long-term potentiation (LTP) and long-term depression (LTD) constitute two forms of synaptic plasticity that are thought to play important roles in learning and memory encoding, as well as the developmental fine-tuning of synaptic connectivity [2–8]. Both LTP and LTD have been observed in a large number of brain regions (e.g., hippocampus, neocortex, amygdala, striatum, and cerebellum) and the induction and maintenance mechanisms have been characterized in great detail [4–7, 9, 10].

Usually, different types of electrical stimulation protocols are used for the induction of either LTP or LTD [11]. While LTP is effectively generated by brief (in the order of seconds) bursts of high-frequency stimulation (HFS; e.g., 100–200 Hz), the induction of LTD typically requires more prolonged (e.g., 10–30 min) trains of low-frequency stimulation (LFS; 1–3 Hz), which has been shown to elicit homosynaptic LTD in the hippocampal CA1 field [12–14], the primary visual cortex (V1) [15, 16], and the amygdala [17], among others (for reviews, see [6, 7, 11]).

It is important to note, however, that the effectiveness of LFS to induce LTD appears to depend on several factors, including the age of the animal [18, 19] and the type of experimental preparation used (i.e.,* in vitro* versus* in vivo*). The initial studies showing successful LTD induction in V1 (and other areas) all employed* in vitro* slice preparations [12–15, 19]. Interestingly, the ability to elicit LTD is significantly reduced when the same LFS protocols are applied to the intact brain. Jiang et al. [20] used LFS (900 or 1800 pulses at 1 Hz) of either the lateral geniculate nucleus (LGN), cortical white matter, or layer IV of V1 to induce LTD at layer II/III synapses of V1 in intact, anesthetized rats; neither of the two LFS protocols elicited LTD, regardless of stimulation site. Similarly, Hager and Dringenberg [21] examined four different LFS protocols, applied to the LGN, and found that none of them was able to induce LTD in V1 of urethane-anesthetized rats. Even the strongest stimulation protocol used, consisting of 900 bursts (3 pulses/burst at 20 Hz) delivered at 1 Hz, only caused a transient depression for about 20 min following LFS delivery [21]. Similar results have also been obtained in the hippocampal formation [22, 23].

The studies summarized above suggest that forebrain synapses display a relatively high resistance to the induction LTD under* in vivo* compared to* in vitro* conditions. One of several, plausible explanations for this discrepancy lies in the fact that different levels of spontaneous synaptic and/or spiking activity influences the effectiveness of LFS to alter synaptic connectivity, as recently proposed by Benuskova [24]. Spontaneous neural activity can be expected to differ substantially between various experimental preparations (e.g.,* in vitro*,* in vivo* anesthetized,* in vivo* conscious, and behaving) and might therefore constitute one of the factors that account for the difference in LTD induction seen in prior work.

Here, we examined if manipulating the levels of spontaneous, cortical activity alters the probability and/or magnitude of LTD at V1 synapses induced by LFS of the LGN; as discussed, prior work has demonstrated a high degree of resistance of this fiber system against LTD induction* in vivo* [20, 21]. The experiments were conducted in rats under very deep urethane anesthesia, a condition that reduces spontaneous activity, as indexed by a burst-suppression pattern of electrocorticographic (ECoG) activity and long (up to several seconds) periods to cessation and neuronal firing (“down-states”) at the level of the cortex and LGN [25–28]. To increase background neuronal activity, animals received electrical stimulation of the brainstem pedunculopontine reticular formation (RF), which exerts a powerful activating effect over the forebrain by eliciting generalized ECoG activation and increasing the discharge rates of cortical neurons [29–32].

## 2. Materials and Methods

### 2.1. Animals

Experiments were conducted in accordance with the published guidelines of the Canadian Council on Animal Care and approved by the Queen's University Animal Care Committee. All efforts were made to minimize animal suffering and the number of animals used for these experiments. Experiments were conducted on adult (250–500 g) male Long-Evans rats (obtained from Charles River Laboratories, Inc., St. Constant, Québec, Canada). Rats were housed as groups (up to four rats/cage) in a standard colony room (12 : 12-hour reverse light cycle, lights on at 19:00) and had free access to food and water.

### 2.2. Surgery

The surgical and electrophysiological procedures were carried out under deep urethane anesthesia (a total dose of 2.0 g/kg, which was administered intraperitoneally [i.p.] as four 0.5 g/kg doses every 15–20 min, supplemented as necessary; urethane obtained from Sigma-Aldrich, Oakville, Ontario, Canada). Following anesthesia induction, the animal was mounted in a stereotaxic apparatus (David Kopf, Tujunga, CA, USA) and the local anesthetic Marcaine (Hospira Healthcare Corporation, Montreal, Quebec, Canada; 5 mg/kg, administered as 2-3 subcutaneous [s.c.] injections) was applied to the scalp 15 min prior to the start of the surgery. Throughout all procedures, body temperature was monitored and maintained at 36-37°C with an electric heating blanket. Furthermore, visual stimulation was reduced by placing hemostats across the closed lids of both eyes.

For the surgery, an incision was made to expose the skull and small skull holes were drilled above the following brain areas (all measurements in mm from bregma point): V1 (anterior-posterior [AP] −7.5, lateral [L] +3.6); LGN (AP −4.1, L +4.1); and pedunculopontine tegmental RF (AP −8.0, L −2.0). Two additional holes were drilled in the skull over the prefrontal cortex to place a ground and reference connection (jewellery screws attached to miniature connectors). A concentric bipolar stimulation electrode (SNE-100, Rhodes Medical Instruments, David Kopf, Tujunga, CA, USA) was then lowered into each the LGN (ventral [V] −4.8 to −5.1 mm from the skull surface) and the RF (V −7.0). Furthermore, a monopolar recording electrode (125 *μ*m diameter Teflon-insulated steel wire) was placed in the superficial layers of V1 (V −0.8 to −1.2). Final, ventral depths of the LGN and V1 electrodes were adjusted to yield fPSP recordings in V1 with maximal amplitude in response to single-pulse (0.2 ms duration) LGN stimulation.

### 2.3. Electrophysiology

Stimulation of the LGN and RF (0.2 ms pulses for both) was provided by connecting the electrodes to separate stimulus isolation units delivering constant current output (LGN stimulation: ML 180 Stimulus Isolator controlled by a PowerLab 4/25T system, ADInstruments, Toronto, Ontario, Canada; RF stimulation: Model 2100 Isolated Pulse Stimulator, A-M Systems, Inc., Carlsborg, WA, USA). The fPSPs (evoked by single-pulse LGN stimulation) and spontaneous background ECoG activity in V1 were differentially recorded (using the same V1 electrode) against the reference connection in the bone over the prefrontal cortex. Both signals were amplified (BioAmp, ADInstruments), digitized (10 kHz for fPSP, 100 Hz for ECoG), and stored for subsequent offline analysis using Scope (v. 4.1.4; ADInstruments) and Chart (v. 5.5.6) software for fPSPs and EEG, respectively.

### 2.4. Data Collection

Following final electrode adjustments, each rat was left to stabilize for approximately 20 min. Subsequently, an input-output curve was established by stimulating the LGN at intensities of 0.1–1.0 mA (in 0.1 mA increments) and the stimulation intensity found to produce 50–60% of the maximal fPSP amplitude was used for the remainder of the experiment. The fPSPs in V1 were then recorded every 30 s until a stable baseline (30 min) was established (between 95% and 105% of average fPSP amplitude over three consecutive 10-minute intervals). Following baseline recordings, one group (LFS group, *n* = 8) of animals received LFS of the LGN, consisting of 900 stimulation bursts repeated at 1 Hz, with each burst consisting of 3 pulses delivered at 20 Hz. Previous work has shown that this protocol is effective in inducing transient depression of synaptic strength between the LGN and V1 in urethane-anesthetized rats [21]. A second group of rats (LFS + RF group, *n* = 8) received LFS (the same as above), paired with stimulation of the RF (900 bursts, with each burst consisting of 25 pulses at 100 Hz; always 0.3 mA intensity and 0.2 ms pulse duration; 10 ms latency between first LGN and first RF pulse). Three additional groups received LFS + RF stimulation and were given one of the following drug treatments: (a) scopolamine (1 mg/kg, *n* = 6); (b) mecamylamine (0.5 mg/kg, *n* = 6); or (c) scopolamine + mecamylamine (10 and 5 mg/kg, respectively; *n* = 4). All drugs were administered i.p., 20 min prior to the onset of baseline fPSP recordings. A final group (RF group, *n* = 6) received RF stimulation (the same as above) without LFS of the LGN. Following delivery of the stimulation protocol, single-pulse stimulation of the LGN resumed and fPSP were recorded (every 30 s) for another 90 min.

### 2.5. Histology

Upon completion of the experiment, animals were perfused through the heart with 0.9% saline (~50 mL), followed by a 10% formalin solution (~100 mL). The brains were harvested and stored for a minimum of 24 h in 10% formalin, after which they were sectioned via a cryostat (40 *μ*m slices). The sections were mounted on glass slides and then used to verify electrode placements, using a digital microscope and rat brain atlas [33]. The accuracy of placements was determined by an experimenter who was blind to the experimental results. Data from inaccurate placements were omitted from the results.

### 2.6. Data Analysis

The fPSPs in V1 were analyzed using Scope software (v. 4.1.4, AD Instruments). The amplitude of the negative peak of the fPSP was computed offline by calculating the voltage difference between the activity immediately prior to the stimulus artefact and that of the maximum peak negativity. These amplitude values were then averaged over 10-minute intervals and normalized by dividing them by the average baseline amplitude of each animal.

The ECoG recorded in V1 was analyzed offline using Chart software (v. 5.5.6, AD Instruments). Two 30-second epochs were analyzed, one immediately prior to the onset of LFS delivery (baseline ECoG) and one during LFS delivery (halfway during the 15 min LFS protocol). The raw ECoG signal was band-pass filtered (0.1 to 5 Hz) to attenuate stimulation-related, high-frequency artifacts in the recording. The filtered ECoG signal was then subjected to power spectral analysis using Chart software (Fast-Fourier Transformation, size 512, Cosine-Bell function applied).

All data are expressed as mean ± standard error of the mean (SEM). Statistical comparisons were made using mixed-model analyses of variance (ANOVA) and, where statistically appropriate, pairwise post hoc comparisons using the SPSS software package (version 21.0, SPSS Inc., IL, USA).

## 3. Results

### 3.1. LFS and LFS + RF Stimulation

**Figure 1:**
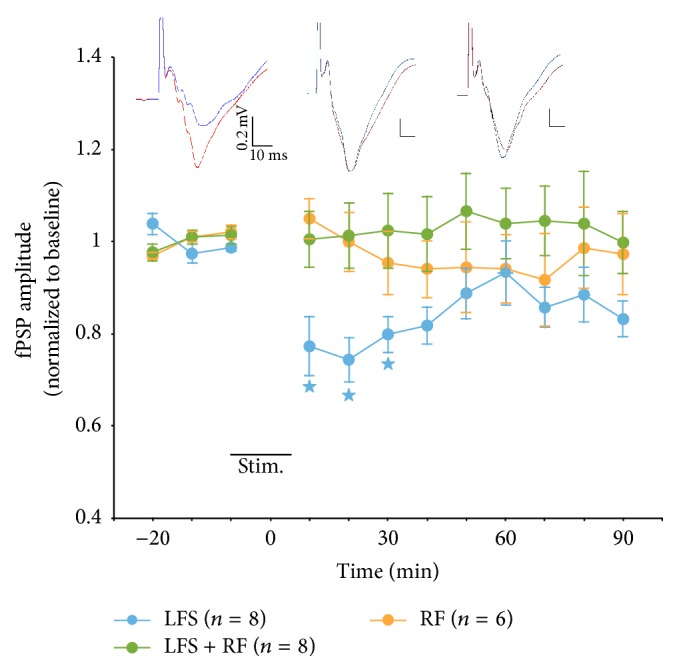
Amplitude of fPSPs recorded in V1 in response to single pulse stimulation of the LGN in urethane-anesthetized rats. Application of LFS (stim.) to the LGN resulted in significant depression of fPSP amplitude. The effect of LFS to elicit LTD was blocked in rats that received paired LFS + RF stimulation. Stimulation of the RF alone did not result in any significant changes in fPSP amplitude. The inserts depict fPSPs recorded before (red) and after (blue) application of the respective stimulation protocol (left: LFS; middle: LFS + RF; right: RF). Asterix (*∗*) indicates significant (*P* < 0.05) difference between LFS and LFS + RF groups.

Single-pulse stimulation of the LGN consistently evoked fPSPs in the superficial layers of the ipsilateral V1, with fPSPs consisting of a large amplitude (up to 0.5 mV) negative component with a latency to peak of about 15–18 ms (Figure 1, insert). These fPSP characteristics are consistent with those reported in previous work using similar electrode configurations [21, 34].

**Figure 2:**
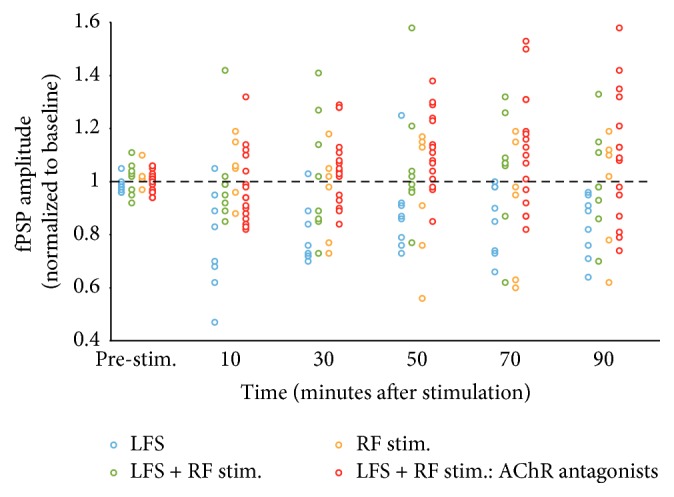
Plot of fPSP amplitude (normalized to baseline) in V1 of individual rats at six time points throughout the experiment. Application of LFS (*n* = 8) to the LGN resulted in significant depression of fPSP amplitude in the large majority of animals (7/8 or 8/8, depending on the time following stimulation). Combined LFS + RF stimulation (*n* = 8), RF stimulation alone (*n* = 6), or LFS + RF stimulation in rats pretreated (i.p.) with acetylcholine receptor (AChR) antagonists (*n* = 16) resulted in heterogeneous responses, with about half of the rats in each group exhibiting potentiation, while the remaining ones showed depression of fPSP amplitude at most time points following delivery of the stimulation protocol (the AChR antagonist condition is comprised of 1 mg/kg scopolamine, *n* = 6; 0.5 mg/kg mecamylamine, *n* = 6; 10 mg/kg scopolamine + 5 mg/kg mecamylamine, *n* = 4; total *n* = 16; see text and Figure 3 for details).

Application of LFS (LFS group, *n* = 8; LFS consisting of 900 stimulation bursts repeated at 1 Hz; each burst containing 3 pulses delivered at 20 Hz) resulted in a significant reduction of fPSP amplitude, with normalized (to baseline) amplitude decreasing to 0.74 (i.e., 74% of baseline) 20 min after LFS delivery (Figure 1). Subsequently, fPSP amplitude showed a gradual increase but remained below baseline levels throughout the entire 90 min recording period after LFS application, with normalized fPSP amplitude at 0.8, 0.93, and 0.83 of baseline levels at 30, 60, and 90 min following LFS delivery (Figure 1). The mean normalized amplitude of the LFS group over the entire 90 min period after LFS delivery was 0.84 (i.e., 84%) of baseline amplitude, indicative of successful LTD induction in this group of animals (Figure 1). This interpretation was supported by an ANOVA, which revealed a significant effect of time on fPSP amplitude in rats receiving LFS, *F*(11,77) = 4.9, *P* < 0.001. Furthermore, at the end of the experiment (i.e., 90 min after LFS), fPSP amplitude was still significantly suppressed relative to all three baseline values (*P* < 0.05, pairwise comparisons), even though there also was one post-LFS time point (60 min after LFS) where amplitude no longer differed from any of the baseline fPSP amplitude values (Figure 1).  Figure 2 depicts normalized fPSP amplitude of individual animals at six time points throughout the experiment. Note the depression of fPSP amplitude in most rats receiving LFS immediately (first 10 min) after stimulation (Figure 2). Furthermore, depression was maintained for the large majority of rats (7/8 at 10, 30, 50, and 70 min after LFS; 8/8 at 90 min after LFS) throughout the entire recording period after LFS application (Figure 2).

A second group of rats (LFS + RF group, *n* = 8) received LFS, paired with stimulation of the pedunculopontine tegmental area of the RF (900 bursts, each burst consisting of 25 pulses at 100 Hz, 0.3 mA intensity, and 10 ms latency between LGN and RF bursts). Surprisingly, in this group, fPSPs did not show any evidence of synaptic depression, with normalized fPSP amplitude in the range of 0.99 to 1.07 (mean of 1.03) of baseline levels over the 90 min recording period following LFS + RF stimulation (Figure 1). An ANOVA comparing the LFS and LFS + RF groups revealed a significant main effect of group, *F*(1,14) = 5.6, *P* = 0.033, as well as a significant group × time interaction, *F*(11,154) = 2.3, *P* = 0.043 (Huynh-Feldt correction applied), but no main effect of time, *P* = nonsignificant (NS). These statistical results confirm that LFS-induced LTD was reversed by combined LFS + RF stimulation. Inspection of individual animals showed that about half of the rats receiving LFS + RF stimulation showed some depression, while the other half exhibited an increase in fPSP amplitude over the 90 min after simulation (Figure 2).

The apparent block of LTD induction by pairing LFS with RF stimulation could be due to synaptic potentiation elicited by the RF stimulation protocol. This possibility was examined by testing a group of rats (RF group, *n* = 6) that received RF stimulation (the same protocol as above) without LFS applied to the LGN. In these animals, fPSPs remained stable over the entire recording period, with normalized fPSP amplitude fluctuating in the range of 0.92 to 1.05 (mean of 0.97) of baseline over the 90 min following RF stimulation (Figure 1). An ANOVA comparing the LFS + RF and RF groups did not reveal a significant group effect, *F*(1,12) = 0.3,* P* = NS, or group × time interaction,* P* = NS, providing further confirmation that LFS was ineffective when combined with RF stimulation. Plots of individual animals showed that, at most time points, about half of the rats in this group showed depression, while the other half exhibited facilitation of fPSP amplitude (Figure 2). Together, these observations indicate that RF stimulation blocks LFS-induced LTD and that this effect is not due to synaptic enhancement elicited by RF excitation.

### 3.2. Blockade of Muscarinic and Nicotinic Receptors

**Figure 3:**
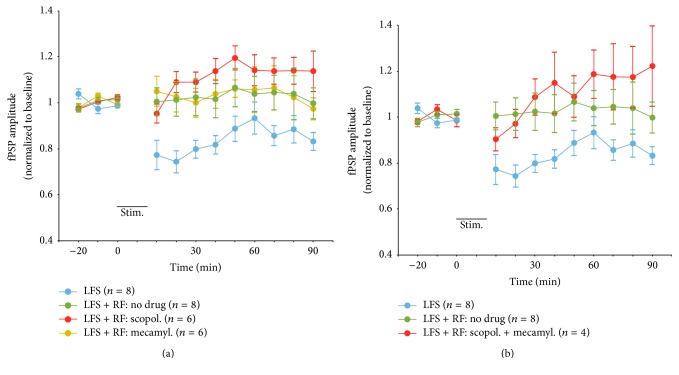
Amplitude of fPSPs recorded in V1 in response to single pulse stimulation of the LGN in urethane-anesthetized rats. (a) Application of LFS (stim.) to the LGN resulted in significant depression of fPSP amplitude (the same group as in Figure 1), an effect that was blocked by pairing LFS with RF stimulation (LFS + RF; the same group as in Figure 1). The effect of RF stimulation to block LTD was not affected by administering (20 min prior to the onset of baseline recordings) either scopolamine (scopol., 1 mg/kg, i.p.) or mecamylamine (mecamyl., 0.5 mg/kg, i.p.). (b) A combination of both drugs at very high doses (scopol., 10 mg/kg + mecamyl., 5 mg/kg) also failed to reverse the block of LTD elicited by pairing LFS with RF stimulation.

One of the major, neurochemical systems controlled by the RF is the cholinergic neurons of the brainstem and basal forebrain [30], with RF stimulation eliciting widespread release of acetylcholine (ACh) from the cortical mantle [35–37]. Thus, it is possible that the effect of RF stimulation to block LTD in V1 following thalamic LFS is due to the release for ACh. This hypothesis was tested by treating separate groups of rats with either the muscarinic receptor antagonist scopolamine (1 mg/kg, i.p.; *n* = 6), the nicotinic receptor antagonist mecamylamine (0.5 mg/kg, i.p.; *n* = 6), or a combination of these two drugs at very high doses (10 mg/kg scopolamine + 5 mg/kg mecamylamine, i.p.; *n* = 4; all drugs given 20 min prior to the onset of baseline recordings). Surprisingly, rats given either scopolamine or mecamylamine still showed the blockade of synaptic depression elicited by paired LFS-RF stimulation (Figure 3(a); normalized amplitude means over 90 min following stimulation of 1.11 and 1.03 for the scopolamine and mecamylamine group, resp.). In fact, RF stimulation continued to block synaptic depression even when the two drugs were administered concurrently at much (10x) higher doses (Figure 3(b); amplitude mean of 1.11). Thus, it appears unlikely that the lack of drug effectiveness was due to the use of insufficient doses or some compensatory interaction between muscarinic and nicotinic receptors. Separate ANOVAs did not reveal any significant group effects when the LFS + RF (no drug) group was compared with LFS + RF rats receiving scopolamine, *F*(1,12) = 0.85,* P* = NS, mecamylamine, *F*(1,12) = 0.003,* P* = NS, or a combination of the two drugs, *F*(1,10) = 0.42,* P* = NS. These data suggest that muscarinic and nicotinic receptor activation is not necessary for the effect of RF stimulation to block the induction of LTD in V1 by thalamic LFS. Individual animals are plotted in Figure 2, which shows that there was a trend for the majority of rats to exhibit increases in fPSP amplitude over the course of the experiment.

### 3.3. EEG Effects

**Figure 4:**
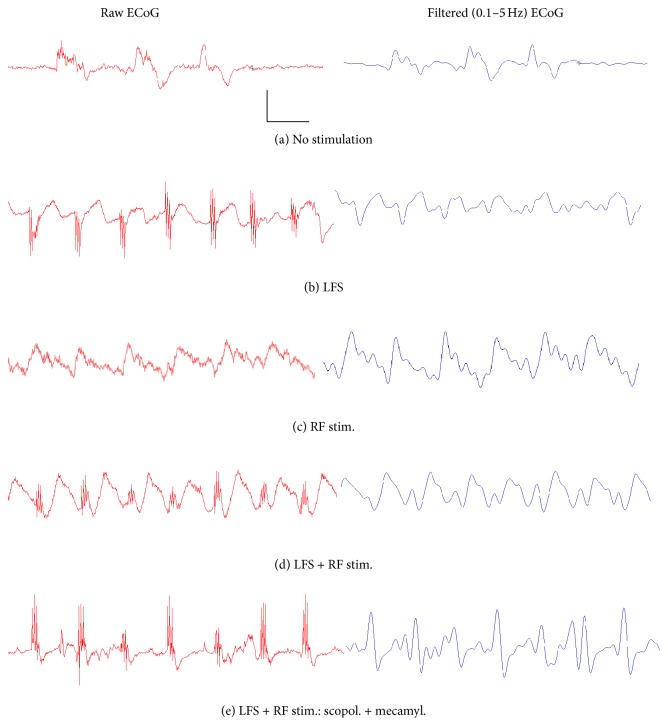
Examples of unfiltered and band-pass filtered (0.1–5 Hz) ECoG activity recorded in V1 of urethane-anesthetized rats. (a) Baseline (prestimulation) ECoG activity showed large, slow oscillations at very low frequencies (<1 Hz) and periods of electrical quiescence. (b) Application of LFS to the LGN resulted in the appearance of more regular, continuous activity between 0.6 and 2 Hz. (c) RF stimulation alone or (d) paired LFS + RF stimulation resulted in a further increase in the frequency and amplitude of cortical activity. (e) Rats treated with a combination of scopolamine and mecamylamine (scopol., 10 mg/kg + mecamyl., 5 mg/kg) continued to exhibit a more complex, rhythmic activity during LFS + RF stimulation (calibration bars are 0.5 mV and 1 s).

**Figure 5:**
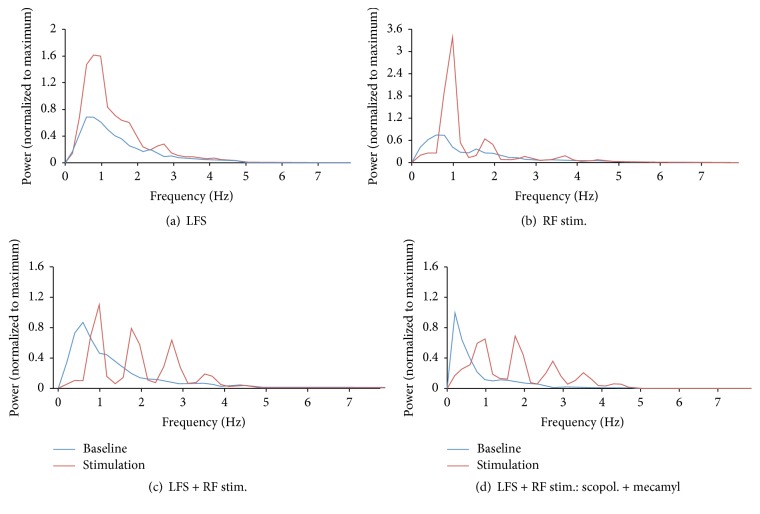
Averaged power spectra for ECoG activity (band-pass filtered between 0.1 and 5 Hz) recorded in V1 of urethane-anesthetized rats. (a) Baseline (prestimulation) ECoG activity showed prominent slow wave activity with peak power between 0.6 and 0.8 Hz. Application of LFS to the LGN enhanced power between 0.6 and 2 Hz. (b) RF stimulation alone or (c) paired LFS + RF stimulation suppressed very low-frequency (~0.6 Hz) power, strongly enhanced power around 1 Hz, and resulted in the appearance of power peaks at ~1.8, 2.7, and 3.5 Hz. (d) Rats treated with a combination of scopolamine and mecamylamine (scopol., 10 mg/kg + mecamyl., 5 mg/kg) continued to exhibit the novel power peaks elicited by LFS + RF stimulation. Power spectra are group averages (*n*'s for all groups, the same as above) and were calculated for 30-second epochs recorded prior to and during delivery of the stimulation protocol. For each rat, power was normalized to the peak power present in the baseline ECoG epoch.

To verify that LFS and pedunculopontine tegmental RF stimulation altered ongoing, cortical activity, we also analyzed the ECoG recorded locally in V1. Under deep urethane anesthesia, the ECoG was dominated by large amplitude (up to 1 mV), slow oscillations (Figure 4(a)). Often, these slow oscillations were interrupted by longer (up to 1 s) periods of strongly suppressed activity, as indicated by flat, isoelectric signals in the ECoG (Figure 4(a)). Power spectral analysis revealed that the peak power was concentrated in the frequency band between 0.6 and 0.8 Hz (Figure 5(a)). During LFS of the LGN, ECoG activity showed more prominent, continuous large amplitude activity and isoelectric periods were abolished (Figure 4(b)). Closer inspection of the ECoG revealed that each stimulation burst evoked a (usually positive-going) wave in the superficial V1. Spectral analyses showed that LFS resulted in an increase in power in the 0.6 to 2 Hz range but also around 2.7 Hz (Figure 5(a)), as well as a slight upward shift in peak power to 0.8 and 1.0 Hz (Figure 5(a)). Similar observations were made in rats that received only RF stimulation (Figure 4(c)), which resulted in a pronounced increase of power around 1 Hz and a concurrent suppression (67%) of very low-frequency power around 0.6 Hz (Figure 5(b)).

Rats that received LFS + RF stimulation showed distinct patterns of ECoG activity during stimulation. Prior to stimulation, these animals also exhibited large, slow activity, with peak power around 0.6 Hz (Figure 5(c)). During stimulation, however, the ECoG switched to a complex, rhythmic activity pattern (Figure 4(d)), with a suppression (of 89%) of the low-frequency (0.6 Hz) power peak and the emergence of a novel, maximal power peak around 1 Hz (Figure 5(c)). Furthermore, additional power peaks emerged at 1.8 Hz, 2.7 Hz, and 3.5 Hz (Figure 5(c)). Thus, combined LFS and RF resulted in the appearance of more complex, rhythmic activity in V1, including a significant amount of activity in higher frequency bands than those normally present under deep, surgical anesthesia.

Interestingly, treatment with either scopolamine, mecamylamine, or both drugs in combination (the same doses as reported above) did not result in a clear attenuation of the evoked activity during LFS and RF stimulation. Scopolamine-treated rats exhibited peak power between 0.4 and 0.6 Hz prior to stimulation (data know shown). During stimulation, this power peak was suppressed by 41% (89% suppression in untreated rats, see above), indicating a reduced effectiveness of stimulation to suppress very low-frequency activity during muscarinic receptor blockade. However, rats still exhibited the appearance of novel power peaks (at about 1 Hz, 1.8 Hz, and 2.7 and 3.5 Hz), similar to the effect seen in untreated animals. Similarly, rats given mecamylamine showed peak power between 0.4 and 0.8 Hz prior to stimulation, which was suppressed by 21% during LFS + RF delivery. Again novel power peaks emerged at the frequencies seen in untreated rats (data not shown). Similar observations were also made in rats that received concurrent treatment of scopolamine and mecamylamine (Figure 4(e)), which also exhibited the characteristic frequency peaks elicited by LFS + RF stimulation (Figure 5(d)).

Together, these data demonstrate that muscarinic and nicotinic receptor blockade reduces the effectiveness of LFS + RF stimulation to suppress very low (<1 Hz) frequency power in the ECoG. However, these drugs do not prevent the emergence of the more complex activity in V1 that results from repeated burst stimulation of the LGN and RF.

## 4. Discussion

The experiments summarized above demonstrate that a strong LFS protocol (see [21]), when applied to the LGN, is effective in inducing LTD in V1 of rats under deep urethane anesthesia. Interestingly, this effect was reversed when LFS was coupled with stimulation of the pedunculopontine tegmentum of the brainstem RF, a major activating centre of the entire forebrain [29–32]. The reversal of LTD was unaffected by high doses of scopolamine and mecamylamine, indicating that cholinergic receptor activation is not required for the effect of RF stimulation to block LTD induction. Background ECoG activity was profoundly altered by LFS + RF stimulation, which caused a suppression of very low (less than 1 Hz) frequency activity and appearance of several, novel peaks in the power spectrum (between 1 and 3.5 Hz). Again, muscarinic and/or nicotinic receptor blockade was not effective in blocking this evoked activity during LFS + RF stimulation, even though these drugs reduced the effectiveness of this stimulation protocol to suppress very low (<1 Hz) frequency power in the ECoG. Together, these data suggest that ongoing, cortical activity may exert an important gating function for plasticity induction at neocortical synapses, with lower activity levels providing a permissive state for the induction of LTD.

Both LTP and LTD are now recognized to play important roles in experience-dependent fine-tuning of synaptic connectivity and information storage in neural circuits [2–8]. In the thalamocortical visual system, LTP and LTD mediate, at least in part, ocular dominance shifts induced by monocular deprivation [8, 38]; the fact that blockade of LTD mechanisms in V1 neurons is sufficient to inhibit ocular dominance plasticity is suggestive of a critical role of LTD in this phenomenon [39]. Surprisingly, however, there are a number of reports of unsuccessful attempt to induce LTD, particularly under* in vivo* conditions [20–23]. For example, Jiang et al. [20] were unable to elicit LTD of field potentials in V1 in rats* in vivo* using a standard LFS protocol (1 Hz stimulation for 15 min) that was highly effective when applied to V1 slice preparations. Similarly, Hager and Dringenberg [21] were unable to induce LTD in V1 of urethane-anesthetized rats using a variety of LTD induction protocols that are effective under* in vitro* conditions.

Several mechanisms have been proposed to account for the resistance of forebrain (hippocampal, neocortical) synapses against the induction of LTD. Jiang et al. [20] found that the presence of brain-derived neurotrophic factor* in vivo* exerts a potent, inhibitory effect on LTD induction in the intact brain. Recently, Benuskova [24], using computational models based on experimental data on LTD induction in dentate gyrus granule cells, suggested that a specific level of ongoing, spontaneous spiking activity is required to elicit LTD, an assumption consistent with experimental observations [40]. Thus, different levels of spontaneous, neural activity may perform important gating functions for the induction of LTD at forebrain synapses.

To examine the role of varying level of spontaneous activity, we deliberately aimed to induce very deep levels of anesthesia, resulting in a burst-suppression pattern of ECoG activity in V1 (see Figure 4). During this state, synaptic and spiking activity is strongly reduced, as indicated by prolonged (up to 1 s) pauses in firing and strongly hyperpolarized (“down-state”) membrane potentials of cortical neurons [25–27]. Under these conditions, LFS was effective in eliciting stable (for >90 min) LTD in V1. Interestingly, the same LFS protocol induced only transient (around 20 min) depression in a prior study [20] that employed a lower dose of urethane than the one used here (2 g/kg versus 1.5 g/kg in [20]). Thus, it is possible that the stronger suppression of cortical activity by a larger anesthetic dose (see [26, 27]) produced a state more conducive to the induction of synaptic depression, even though contributions of other factors (related or unrelated to the level of anesthesia) cannot be excluded.

To further probe this issue, we directly manipulated cortical activity by excitation of the brainstem RF, a major activating system of the forebrain [29–32]. While application of LFS alone strongly enhanced low (~0.3–2 Hz) frequency power, paired LFS + RF stimulation reduced low-frequency power and resulted in the appearance of novel power peaks in the range between 1.8 and 3.5 Hz. Thus, combined LFS and RF stimulation potently altered ECoG activity by concurrently suppressing very slow activity and entraining novel, rhythmic activity in frequency ranges higher than those normally present under deep urethane anesthesia (see present results and [29–32]). Most importantly, thalamic LFS became ineffective in eliciting LTD when it was delivered with RF stimulation. ECoG fluctuations largely reflect synchronized excitatory and inhibitory synaptic currents that summate in the extracellular space and give rise to local field potential that can be detected by extracellular recording electrodes [41–43]. Thus, it appears that, during RF stimulation, the enhanced synaptic activity of cortical networks hinders the induction of LTD in V1 neurons. However, patterns of spiking activity may also play an important role in gating LTD induction, given that spiking activity of neocortical neurons is closely linked to rhythmic oscillations of extracellular (synaptic) currents [41–43].

Given that RF stimulation results in the widespread release of ACh throughout the cortical mantle [35–37], we tested whether blockade of muscarinic and/or nicotinic receptors altered the effect of RF stimulation to block the induction of LTP in V1. Interestingly, neither scopolamine nor mecamylamine, administered alone or together in very high doses, was able to reverse the blockade of LTD induction by RF stimulation. While these results may seem surprising, it is noteworthy that these drug treatments also failed to block the evoked ECoG patterns elicited by LFS + RF stimulation (see Figures 4 and 5), even though both drugs reduced the effectiveness of RF stimulation to suppress very low (<1 Hz) frequency activity, an observation consistent with a role of both muscarinic and nicotinic receptors in ECoG/EEG regulation [44]. Overall, these data strengthen the proposed relation between synaptic activity levels and the probability of LTD induction, in that both the appearance of faster, cortical activity and the inhibition of LTD by RF stimulation were preserved during cholinergic receptor blockade. Furthermore, the observation that drug treatments reduced the suppression of very low (< 1 Hz) frequency power without affecting the blockade of LTD induction suggests that changes in very slow activity may not be a critical contributor to the effect of RF stimulation on LTD induction noted in our experiments.

Our finding that reduced (by deep urethane anesthesia) and enhanced (by RF stimulation) cortical background activity can facilitate and suppress, respectively, synaptic depression is consistent with several investigations. Crochet et al. [45] showed that reduced levels of background activity increased the probability to elicit shorter-term (in the order of minutes) synaptic depression in neocortical networks* in vivo*. Similarly, short-term depression of V1 synapses elicited by paired-pulse stimulation* in vitro* was strongly inhibited when slices were perfused with elevated-potassium medium to elicit spontaneous activity [46]. Together, these data indicate that a state of lowered neural activity may be permissive for the induction of synaptic depression across a variety of time scales (from seconds to hours) in neocortical networks. Furthermore, given that typical slice preparations exhibit relatively little or no spontaneous activity (e.g., [46]), this interpretation can also account for the higher effectiveness of LFS to elicit LTD under* in vitro* compared to* in vivo* conditions, as discussed above. As an important extension of the current experiments, future work could manipulate cortical activity by more naturalistic sensory stimulation, rather than by means of electrical (present study) or chemical manipulations (e.g., [46]). If background activity does, indeed, exert the gating function proposed here, then visual stimuli (e.g., full-field flashes or moving, sinusoidal gratings) and dark exposure should inhibit and facilitate, respectively, LTD induction in V1. At present, this hypothesis remains speculative and requires critical assessment. However, it is worth noting that the eyes of our animals remained closed throughout the experimental procedures, raising the possibility that the lack of visual input contributed to the successful LTD induction in rats that received LFS without coupled RF stimulation.

At the same time, our data and interpretations appear to contradict the model by Benuskova [24], proposing that spontaneous activity is required to elicit LTD. It is important to note, however, that our recordings of V1 activity during delivery of thalamic LFS revealed a pronounced enhancement of synaptic activity in the range of about 0.3–2 Hz compared to spontaneous (pre-LFS) activity. Thus, it is conceivable that this activity level is in the permissive range required to elicit LTD at neocortical synapses. When spontaneous activity is increased (present data) or decreased (see [24]) to levels outside this “permissive window,” LTD is blocked, as seen when LFS was paired with RF stimulation. A possible explanation for this effect might be that synaptic and spiking activity elicited by relatively infrequent (~1 Hz) LFS is now embedded in a more active network. Under these conditions, LFS may lose the ability to relay information about the relative timing of firing of individual pre- and postsynaptic neurons, the main determinant of plasticity induction and direction (i.e., potentiation versus depression), according to spike-time-dependent plasticity (STDP) rules [47–49]. Future experiments that systematically and independently manipulate spontaneous activity of pre- and postsynaptic neurons and assess the consequences on LTD induction are required to assess this hypothesis. The concurrent application of a GABA_A_-receptor agonist and a GABA_B_-receptor antagonist has recently been shown to result in the inhibition of postsynaptic responses, while largely preserving the activity of presynaptic inputs [50, 51]. Thus, this pharmacological approach might be useful in determining the respective roles of spontaneous pre- and postsynaptic firing on LTD induction in thalamocortical sensory (and other) pathways under* in vivo* conditions.

In summary, the present experiments show that stimulation of the brainstem core increases cortical activity and inhibits the induction of LTD in V1. Both effects were resistant to the blockade of cholinergic (muscarinic and nicotinic) receptors. Thus, we propose that increased levels of ongoing, neural activity may limit the ability to elicit depression at cortical synapses.

## Abbreviations

ACh: Acetylcholine
ANOVA: Analysis of variance
AP: Anterior-posterior
ECoG: Electrocorticographic
fPSP: Field postsynaptic potential
HFS: High-frequency stimulation
i.p.: Intraperitoneal
L: Lateral
LFS: Low-frequency stimulation
LGN: Lateral geniculate nucleus
LTD: Long-term depression
LTP: Long-term potentiation
NS: Nonsignificant
RF: Reticular formation
s.c.: Subcutaneous
SEM: Standard error of the mean
V: Ventral
V1: Primary visual cortex.
